# Determining nitrogen losses through breath ammonia production in lactating cows

**DOI:** 10.3168/jdsc.2026-1079

**Published:** 2026-08-24

**Authors:** B. Bae, C. Lee

**Affiliations:** Department of Animal Sciences, The Ohio State University, Wooster, OH 44691

## Abstract

In N balance studies, retained N, calculated by subtracting N secretion and excretion from N intake, has generally been considered to be overestimated in lactating cows. Various studies have investigated potential sources of unaccounted N losses; however, these sources have not been fully identified. In the current study, we evaluated N losses via oral NH_3_-N production, which includes NH_3_-N originating from both eructation and pulmonary exhalation (defined as breath NH_3_-N) as a potential contributor to the unaccounted N fraction in lactating cows. Experiment 1 was conducted to assess breath NH_3_-N production and its diurnal pattern with 8 mid-lactation Holstein cows in a crossover design (21 d per period). Cows received a diet supplemented with SFA or oleic acid (2% of dietary DM). Experiment 2 was conducted to assess the effects of dietary CP level (15.3% vs. 17.0% of dietary DM) on breath NH_3_-N production with 16 mid-lactation Holstein cows in a randomized complete block design (21 d). Multiple breath samples (8 time points and 5 time points in a 24-h cycle during experiments 1 and 2, respectively) were collected from individual cows using a spot breath collection procedure with a facemask system (3-min breath collection for each event). In experiment 1, breath NH_3_-N production (0.82 g/d) was not affected by dietary treatment, and no interaction was observed between diet and collection time in a 24-h feeding cycle. However, breath NH_3_-N production was affected by collection time, indicating a diurnal pattern of breath NH_3_ production. The production decreased immediately after feeding, gradually increased until 6 h after feeding and then remained relatively stable until the next feeding time. In experiment 2, although N intake was greater (802 vs. 701 g/d) for cows fed the high-CP diet compared with the low-CP diet, breath NH_3_-N production did not differ (0.61 g/d) between treatments, resulting in greater breath NH_3_-N production as a proportion of N intake (0.086% vs. 0.075%) for cows fed the low-CP diet. Data from experiments 1 and 2 were combined and analyzed to determine the relationship between N intake and breath NH_3_-N production. The linear regression analysis showed a poor linear association (R^2^ = 0.05; concordance correlation coefficient = 0.11) between N intake and breath NH_3_-N production. In conclusion, N losses in breath were observed, with breath NH_3_ exhibiting a diurnal pattern. However, daily production of breath NH_3_-N was not affected by dietary CP or N intake in the current experiments. Although N losses through breath NH_3_ were measurable, the loss was ignorable because its proportion of N intake was trivial.

## Graphical Abstract

**Figure:**
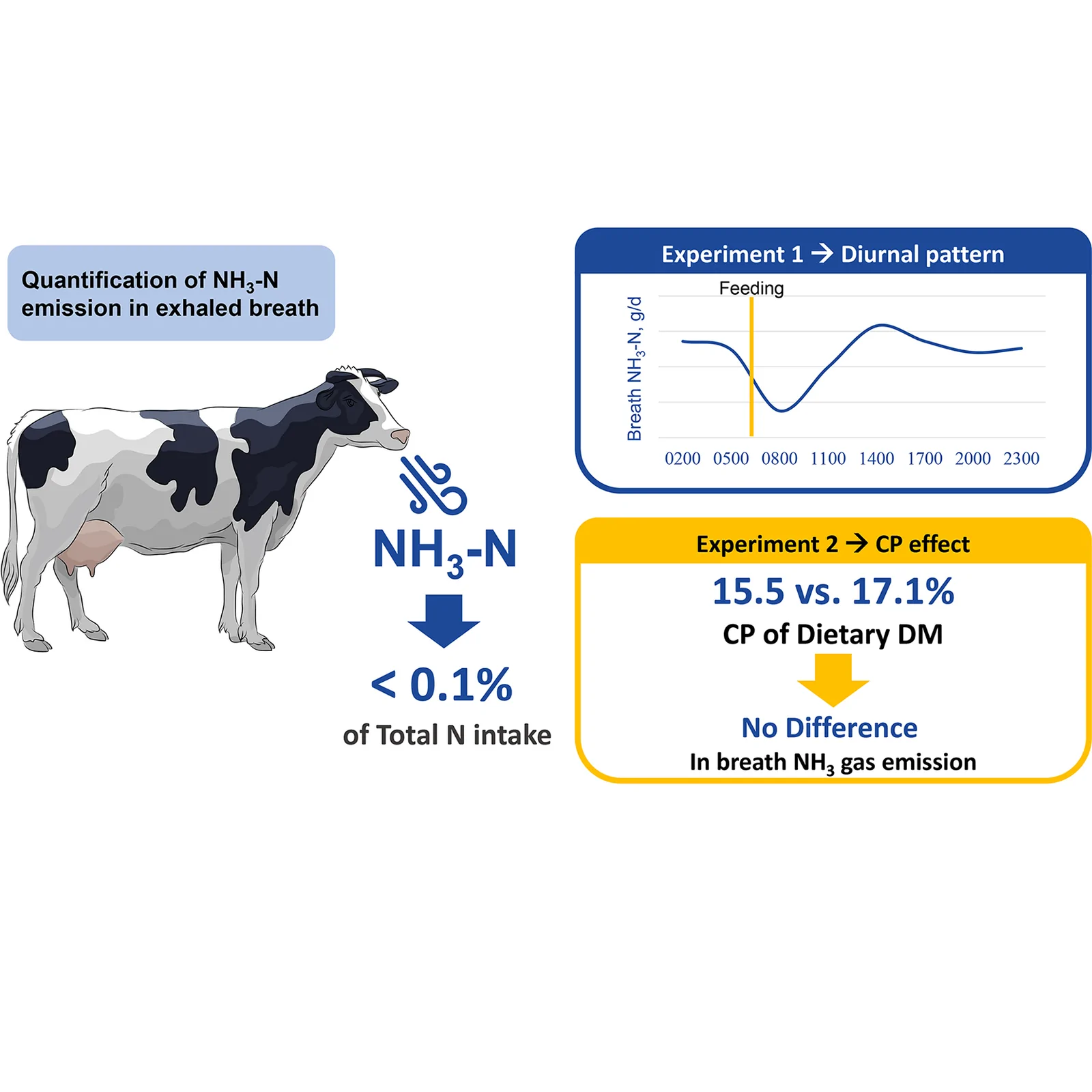

Nitrogen balance has been extensively investigated to evaluate dietary N utilization and excretion in animals (Spanghero and Kowalski, 1997). In dairy cows, N balance provides insight into the gross efficiency in milk protein synthesis, as well as the overall efficiency of protein utilization during lactation (Spanghero and Kowalski, 2021). However, studies in the literature seem to generally overestimate N retention (N intake − N outputs via milk, feces, and urine) in lactating cows by 5 to 10 percentage units when calculated as a proportion of N intake (Spanghero and Kowalski, 1997, 2021). Spanghero and Kowalski (2021) speculated that the overestimation primarily resulted from underestimation of fecal and urinary N excretion, likely due to N losses during sample collection, storage, and analysis. However, the overestimation of N retention does not seem to be fully explained by those factors. It is true that N concentrations in biological samples are affected due to N losses during sample processing and analysis (e.g., drying, analytical methods, and sample preservation; Nichols et al., 2025). However, despite efforts to account for those factors, Nichols et al. (2025) reported that those were insufficient to explain the excess N loss in a balance study conducted using a respiration chamber system. Therefore, there are likely other sources of N loss contributing to the overestimation of N retention.

Ammonia is produced in the rumen through microbial degradation of N compounds or microbial hydrolysis of urea (Abdoun et al., 2006). A proportion of NH_3_ in the rumen can be volatilized and exists in the gaseous phase of the rumen headspace (Dewhurst et al., 2001), suggesting that NH_3_ in the rumen can be lost through eructation. Furthermore, a portion of blood NH_3_^+^ can diffuse into the lung and be excreted in exhaled breath in humans (Neri et al., 2012). Therefore, there is potential that N can be lost through NH_3_ from eructation and the lungs, which may, at least in part, contribute to unaccounted N losses in ruminants. To our knowledge, daily losses of orally produced NH_3_-N that originates from both eructation and the lungs (hereafter defined as breath NH_3_-N) have not been quantified and studied as a source of N loss. Assuming a ruminal NH_3_-N concentration of 10 mg/dL (19.5 ± 2.7 kg/d DMI, 27.2 ± 7.0 kg/d milk yield, 624 ± 65 kg BW; Huhtanen et al., 2015) and a rumen volume of 120 L (Melgar et al., 2020), the ruminal NH_3_-N pool would be ∼12 g. If 0.1% of the ruminal NH_3_-N pool is volatilized and lost via eructation at a rate of 0.86 events/min in lactating cows (Wang et al., 2023), this would correspond to a N loss of ∼15 g/d as breath NH_3_, highlighting its potential as an unaccounted source of N loss. Nevertheless, the assumption of negligible N loss through NH_3_ in breath may be valid because rumen pH is relatively low, resulting in a small proportion of NH_3_ relative to NH_4_^+^ in the NH_4_^+^/NH_3_ equilibrium (Abdoun et al., 2006) and, consequently, limited NH_3_ volatilization. However, NH_3_ volatilization is affected by various factors. For example, although manure pH ranges from 6.0 to 7.5, considerable amounts of NH_3_ are volatilized from manure (Dewes, 1996). If NH_3_ volatilization in the rumen exists (Dewhurst et al., 2001) and is affected by factors other than rumen pH, it is possible that N can be lost through eructation and potentially be a source of unaccounted N in a N balance study. The objective of this study was to quantify breath NH_3_ production as a potential source of N losses in lactating cows. We hypothesized that breath NH_3_ production may contribute to unaccounted N losses, which, at least in part, explains the overestimation of N retention that is often observed in studies with lactating cows.

All experiments were conducted at the Krauss Dairy Research Center (The Ohio State University, Wooster, OH), and all procedures involving animals in this study were approved by The Ohio State University Institutional Animal Care and Use Committee (2024A00000090 and 2024A00000029). Experiment 1 was a part of a larger experiment involving 8 mid-lactation Holstein cows (average ± SD; 194 ± 21 DIM, 46 ± 5 kg/d milk yield, 687 ± 51 kg BW) in a crossover design. The objective of the larger experiment was to assess the effects of feeding different fatty acids (**FA**) on lactation performance and methane production. The current experiment focused on investigating the diurnal pattern of breath NH_3_-N production. Cows were assigned to 4 squares based on parity and DIM, with 2 cows each. Within each square, cows were randomly assigned to a diet supplemented with SFA (22.1% of C16:0, 20.0% of C18:0, and 12.0% of *cis*-9 C18:1 in dietary FA) or with oleic acid (11.3% of C16:0, 3.2% of C18:0, and 38.0% of *cis*-9 C18:1 in dietary FA). Both diets were composed of 39.4% of corn silage, 14.3% of alfalfa silage, and 46.3% of concentrate on a DM basis (16.2% of CP, 30.2% of NDF, 28.0% of starch, and 4.5% of FA on a DM basis). Diets were offered once daily (0700) for ad libitum intake. Each period lasted for 21 d, and breath collection was conducted from individual cows during the last 3 d in each period. Breath collection was conducted using a facemask system as described in Bae et al. (2026). Briefly, the facemask system generates one-way flow in the system at 250 L/min at standard temperature and pressure (**STP**; 0°C, 101.3 kPa), and the air flow sweeps entire breaths from the cow wearing the mask. The air flow is measured in real time and sampled continuously at 0.6 L/min into a gas sampling bag (Tedlar Sampling Bag 22501, Restek Co.) during breath collection. Spot breath collection was conducted for individual cows 8 times over 3 d to represent every 3-h sampling in a 24-h cycle (Hristov et al., 2015). At each time point, the facemask system was applied to each cow and breath samples were collected for 3 min. The collected air samples were analyzed for NH_3_ concentration using a photoacoustic gas analyzer (GASERA ONE, Gasera Ltd.). The following equation was used to calculate daily production of NH_3_-N at each time point:

Breath NH_3_-N production (g/d) = NH_3_ concentration × air flow (L/min) at STP × NH_3_ density (0.769 g/L at STP) × 1,440 × 14/17 (molecular weights of N and NH_3_).

Experiment 2 was conducted to assess the effects of dietary CP level on breath NH_3_-N production with 16 mid-lactation Holstein cows (average ± SD; 145 ± 34 DIM, 51 ± 7 kg/d milk yield, 700 ± 57 kg BW) in a randomized complete block design. Cows were blocked by parity and DIM. Within each block, cows were randomly assigned to varying CP diets (15.3% or 17.0% CP of DM). Both diets were composed of 34.6% corn silage, 22.0% alfalfa silage, and 43.4% concentrate on a DM basis. The low-CP diet contained 15.3% CP, 28.3% NDF, 26.2% starch, and 4.7% FA on a DM basis, and the high CP diet contained 17.0% CP, 28.5% NDF, 27.0% starch, and 4.6% FA on a DM basis. All diets were offered once daily (0700 h) for ad libitum intake. The experiment consisted of 18 d of diet adaptation and 3 d of breath collection. Breath samples were collected at 5 time points over 3 d (1, 6, 9, 15, and 18 h after feeding) and daily NH_3_-N productions were determined as described above. One cow fed the high-CP diet was a cannulated cow and showed considerably lower breath NH_3_-N production, probably due to a gas leak through cannula (Wang et al., 2019). Therefore, all data from that cow were excluded and treated as missing data. During both experiments, feed offered and refused were recorded daily and their DM was determined for DMI (method 934.01, AOAC International, 2000). Feed samples were analyzed for total N using a combustion method (Flash 2000, Thermo Fisher Scientific), and N intake for individual cows were calculated.

All data were analyzed using PROC MIXED of SAS (version 9.4, SAS institute Inc.). The data of breath NH_3_-N production from individual collection times in experiment 1 (n = 128) were analyzed using a model with period and cow within square as random effects and diet, breath collection time, and their interaction as fixed effects. The data of breath NH_3_-N production from individual cows in experiment 2 (n = 15 after excluding one cannulated cow) were analyzed using a model with block as a random effect and dietary CP level as a fixed effect. The breath NH_3_-N production from individual time points in experiment 1 were averaged by cow and period (n = 16) and were combined with the data from experiment 2. The combined data of daily NH_3_-N production (mg/d) from experiments 1 and 2 (n = 31) were analyzed for a linear relationship with N intake (independent variable) and breath NH_3_-N production (dependent variable), where experiment was included as a random effect. In addition to the linear regression analysis, the concordance correlation coefficient (**CCC**; Lin, 1989) was calculated to assess the correlation between N intake and breath NH_3_ production. All data were presented as LSM, and mean differences were considered significant when *P* < 0.05 and a tendency when 0.05 ≤ *P* < 0.10.

In experiment 1, breath NH_3_-N production (0.68 g/d, SEM = 0.050) was not affected by dietary treatments and had no interaction of diet by collection time (Figure 1). However, breath NH_3_-N production was affected (*P* < 0.01) by collection time and showed a diurnal pattern. Breath NH_3_-N production decreased immediately after feeding and gradually increased until 7 h after feeding. Thereafter, there was no difference in breath NH_3_-N production from 10 to 22 h after feeding. Ammonia is a weak base with p*K*_a_ of 9.0 (Leng and Nolan, 1984). Therefore, most ammoniacal N in the rumen (pH 5.5–6.5) exists in its ionized form, ammonium (NH_4_^+^), according to the Henderson–Hasselbalch equation (Abdoun et al., 2006). Rumen pH typically declines after feeding due to feed fermentation and VFA production, after which it gradually increases until the next feeding (Rustomo et al., 2006). For volatilization, ammoniacal N should exist as NH_3_ in rumen fluid. According to the Henderson–Hasselbalch equation, the proportion of NH_4_^+^ and NH_3_ in fluid is affected by pH. Although ruminal pH was not measured in the current study, lowering ruminal pH after feeding is expected (Argyle and Baldwin, 1988), which theoretically decreases the proportion of NH_3_ in ruminal ammoniacal N, resulting in less NH_3_ exhaled through eructation. In addition, although the fraction is small, NH_3_ in breath can originate from the lungs according to a human study (Neri et al., 2012). Ammonium needs specific carriers or channels to be absorbed from the rumen, whereas NH_3_ easily diffuses across the rumen epithelium (Abdoun et al., 2006). Thus, lowering rumen pH decreases the proportion of NH_3_ in ruminal ammoniacal N and decreases overall absorption of ammoniacal N across the rumen epithelium into the blood (Kertz et al., 1983). This probably resulted in decreases in diffusion of NH_3_ into the lung, and subsequently NH_3_ exhaled through breaths. Accordingly, the diurnal pattern of NH_3_-N production, which was similar to the diurnal pattern of rumen pH under a once-a-day feeding conditions, seems reasonable.

**Figure 1:**
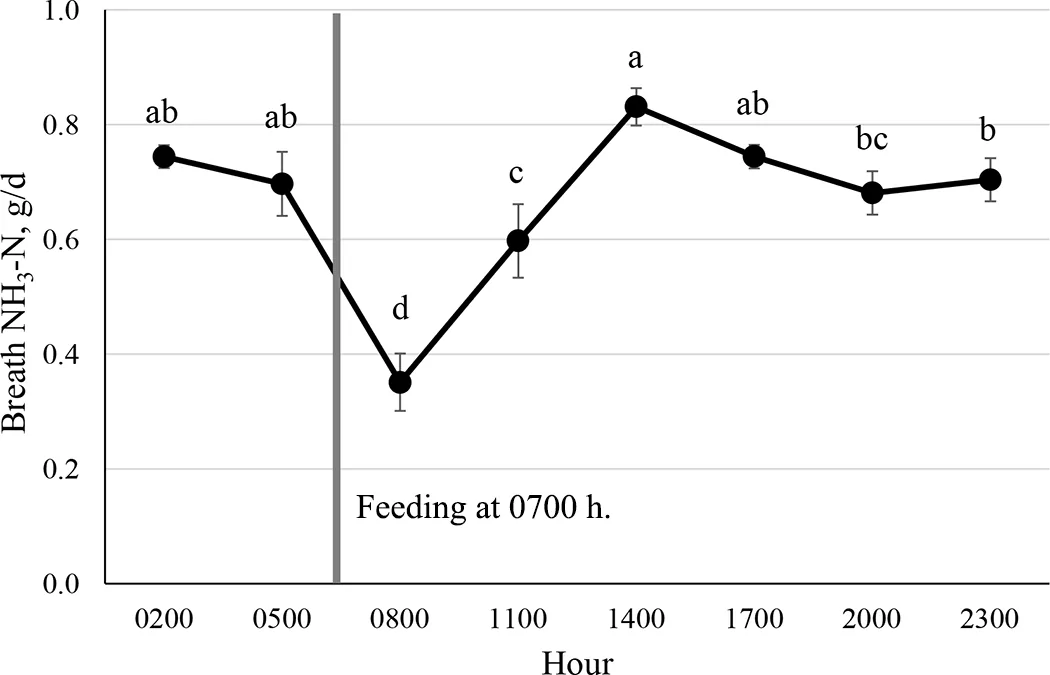
The diurnal pattern of breath NH_3_-N production (g/d) in experiment 1. Dietary treatment, *P* = 0.88; collection time, *P* < 0.01; interaction between diet and time, *P* = 0.98. Error bars = SE within time point. Among time points, means without a common lowercase letter differ (*P* < 0.05).

In experiment 2, N intake was greater (802 vs. 701 g/d; *P* < 0.01) for cows fed the high-CP diet compared with the low-CP diet (Table 1). Despite the difference in N intake, breath NH_3_-N production did not differ (0.61 g/d) between dietary treatments. As a result, breath NH_3_-N as a proportion of N intake was greater (*P* < 0.01) for cows fed the low-CP diet compared with cows fed the high-CP diet. An increase in dietary CP concentration often increases ruminal NH_3_ concentrations (Roffler and Satter, 1975). The lack of difference in breath NH_3_-N production between the diets suggests that NH_3_ volatilization in the rumen is likely regulated by factors other than N intake. Dewhurst et al. (2001) found that rumen pH has a greater effect on gaseous NH_3_ concentration compared with concentration of ammoniacal N in the rumen fluid. In that study, the gaseous NH_3_ concentration increased as rumen pH increased, although ammoniacal N in the fluid declined. This indicates that ruminal pH may play a primary role in regulating NH_3_ volatilization in the rumen regardless ruminal ammoniacal N concentration. Furthermore, NH_3_ volatilization in the rumen might be influenced by other factors, such as temperature and mixing, similar to NH_3_ volatilization in manure (Van der Stelt et al., 2007). The result of the linear regression analysis supports the lack of difference in breath NH_3_-N production between the high- and low-CP diets (Figure 2); breath NH_3_-N (mg/d) = 0.18 × N intake (g/d; SE = 0.22) + 500.9 (SE = 171.3), where the slope was not different from 0 (*P* = 0.41; R^2^ = 0.05). Furthermore, we observed a poor linear association between N intake and breath NH_3_-N production according to the CCC (0.11).

**Table 1:** Effects of dietary CP level on breath NH_3_-N emission in experiment 2

Item	Diet^1^		SEM	*P*-value
	Low CP	High CP		
N intake, g/d	701	802	19.1	<0.01
Breath NH_3_-N, g/d	0.60	0.61	0.041	0.80
Breath NH_3_-N, % of N intake	0.086	0.075	0.0037	<0.01

1 Low CP = 15.3% of DM; High CP = 17.0% of DM.

**Figure 2:**
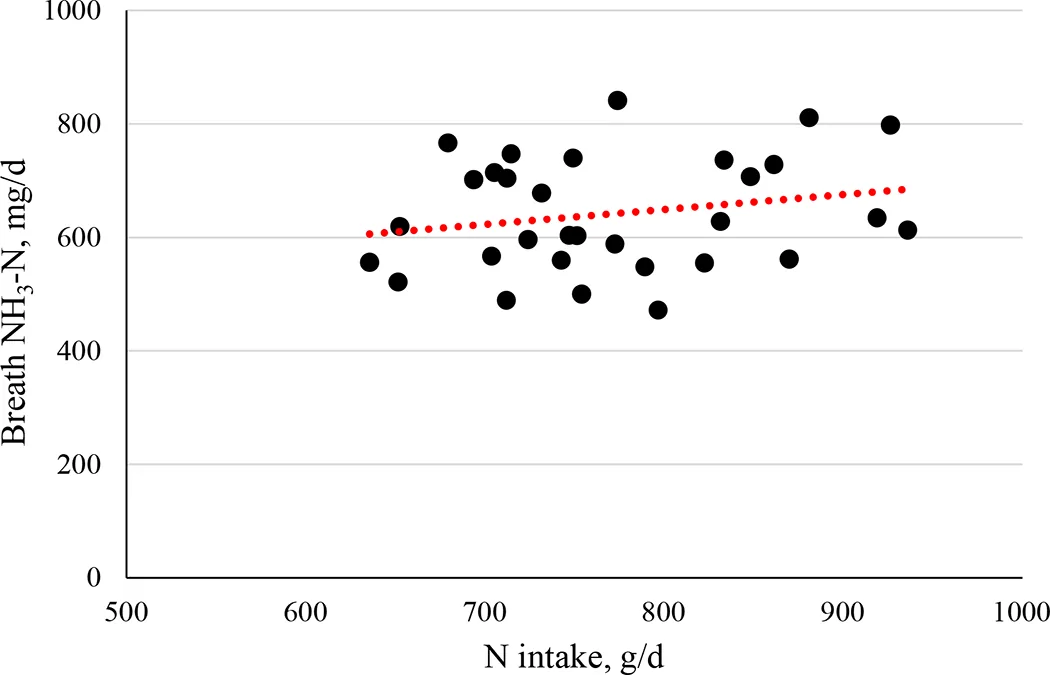
The association between N intake (g/d) and breath NH_3_-N production (mg/d). Breath NH_3_-N (mg/d) = 0.18 × N intake (g/d; SE = 0.22) + 500.9 (SE = 171.3). Regression, *P* = 0.41. R^2^ = 0.05. CCC = 0.11.

Breath NH_3_ has been investigated primarily in human studies, showing a high correlation between BUN and breath NH_3_ (*P* < 0.01; r = 0.77–0.84; Neri et al., 2012). In contrast, to our knowledge, there has been only one study determining NH_3_-N production in breath of ruminants. Valente et al. (2018) measured breath NH_3_ production from Holstein heifers (BW, 267 ± 11 kg) using a facemask system to investigate ruminal N metabolism. In that study, heifers produced ∼8 µg/min/kg^0.75^ of NH_3_ in breath, which corresponds to approximately 0.76 g/d of breath NH_3_ or 0.62 g/d of breath NH_3_-N, and these values are comparable to the breath NH_3_ production observed in the current study. However, in that study, breath NH_3_ increased with increasing dietary CP level (9%, 12%, 15%, and 18% of dietary DM) and showed a linear correlation (*P* < 0.01; r = 0.68) between dietary CP level and breath NH_3_ excretion (µg/min/kg^0.75^), which contrasts with our findings. The discrepancy occurred probably due to different diet composition. In the study by Valente et al. (2018), dietary CP was increased by increasing soybean meal inclusion from 0% to 21.1% of dietary DM while decreasing corn grain from 53.8% to 32.7% of dietary DM. Although the authors did not report dietary starch concentrations, the experimental diets included corn grain as the sole starch source. Based on this, starch concentrations were assumed to be 38%, 33%, 28%, and 23% of dietary DM for the 9%, 12%, 15%, and 18% CP diets, respectively, assuming starch concentration of 72% of DM in corn grain. Therefore, the linear increase in breath NH_3_ production with increases in dietary CP observed in Valente et al. (2018) may have occurred due to rumen pH largely altered by starch intake rather than N intake. In the current experiment, although increasing dietary CP from 15.3% 17.0% decreased dietary starch concentration (27.0% to 26.2% of dietary DM), the difference was small. Nevertheless, we found that N loss as breath NH_3_-N was less than 0.1% of N intake and was negligible as a source of unaccounted N losses contributing to overestimation of N retention in lactating cows.

It is worth noting that NH_3_ is a highly adsorptive gas, and gaseous NH_3_ molecules may adhere to the surfaces of sampling tubes and bags during collection (Sintermann et al., 2012). Although the facemask system used in this study was previously validated for CH_4_ and CO_2_ measurements in lactating cows (Bae et al., 2026), it was not validated for NH_3_. Therefore, breath NH_3_-N production (g/d) and its proportion of N intake may have been underestimated if NH_3_ collected using the facemask system was not fully recovered. However, the continuous air-flux multichamber system for manure incubation to monitor NH_3_ and greenhouse gas emissions (Lee et al., 2020; Zynda et al., 2022; Hu et al., 2026) was constructed using similar components (e.g., pumps, tubing, and collection bags) based on the same principles as the facemask system. In that system, multiple NH_3_ recovery tests resulted in values of 98% to 103% (data not shown). In addition, Akdeniz et al. (2011) evaluated the effects of gas collection bags on NH_3_ recovery and reported 100% to 110% of NH_3_ recoveries when Tedlar sampling bags were used. Therefore, underestimation of breath NH_3_-N due to the adsorptive nature of NH_3_, resulting in incomplete recovery from the sampling system, is unlikely in the current study. Another potential limitation of the current study was the use of spot breath sampling to estimate daily NH_3_-N production. Although the spot sampling approach has been validated for estimating daily CH_4_ and CO_2_ production in lactating cows (Hristov et al., 2015; Bae et al., 2026), it has not been validated for NH_3_-N production. However, in the present study, spot sampling conducted at 3-h intervals over a 24-h period under a once-daily feeding regimen adequately captured the diurnal pattern of breath NH_3_-N production (Figure 1) and seems appropriate. However, further studies are needed to characterize the diurnal variation in breath NH_3_-N production using measurement systems capable of monitoring breath NH_3_-N at shorter sampling intervals, such as respiratory chamber or respiratory head-chamber systems. Lastly, breath NH_3_-N production that was influenced by dietary CP levels and N intake was evaluated in the current study. However, because breath NH_3_-N production is associated with ruminal pH, daily breath NH_3_-N production might be influenced by feeding behavior, feeding frequency, diet composition, and feed intake, which potentially alter ruminal pH and its diurnal pattern (Aschenbach et al., 2011; Dijkstra et al., 2012; Beauchemin, 2018). Although these factors may affect the absolute amount of breath NH_3_-N production, breath NH_3_-N production is likely to remain negligible regardless of those factors due to the low amount of breath NH_3_-N production in the current study.

In conclusion, N loss as breath NH_3_ production was detected in the current study and decreased significantly immediately after feeding, likely due to a decrease in ruminal pH. However, daily breath NH_3_ production was not affected by dietary CP level or N intake under the conditions of the current experiment. Although the loss of N as breath NH_3_ was measurable, it represented a trivial proportion of N intake and therefore was not a source of unaccounted N loss contributing to the overestimation of N balance that is often observed in studies with lactating cows.
